# Chronic Mg^2+^ Deficiency Does Not Impair Insulin Secretion in Mice

**DOI:** 10.3390/cells12131790

**Published:** 2023-07-05

**Authors:** Noushafarin Khajavi, Klea Riçku, Pascale C. F. Schreier, Tanja Gentz, Philipp Beyerle, Emmanuel Cruz, Andreas Breit, Peter S. Reinach, Thomas Gudermann

**Affiliations:** 1 Walther Straub Institute of Pharmacology and Toxicology, LMU Munich, 80539 Munich, Germany; klearicku@gmail.com (K.R.); pascale.schreier@lrz.uni-muenchen.de (P.C.F.S.); t.gentz@campus.lmu.de (T.G.); philipp.beyerle@web.de (P.B.); e.cruz@campus.lmu.de (E.C.); andreas.breit@lrz.uni-muenchen.de (A.B.); 2 Ophthalmology Department, Wenzhou Medical University, Wenzhou 325015, China; preinach25@gmail.com; 3 German Center for Lung Research, 81377 Munich, Germany

**Keywords:** hypomagnesemia, insulin secretion, calcium signaling, type 2 diabetes

## Abstract

Magnesium is an essential mediator of a vast number of critical enzymatic cellular reactions in the human body. Some clinical epidemiological studies suggest that hypomagnesemia accounts for declines in insulin secretion in patients with type 2 diabetes (T2D); however, the results of various experimental studies do not support this notion. To address this discrepancy, we assessed the short- and long-term effects of hypomagnesemia on β-cell function and insulin secretion in primary mouse islets of Langerhans and in a mouse model of hypomagnesemia known as *Trpm6^Δ17 /fl^;Villin1-Cre* mice. We found that lowering the extracellular Mg^2+^ concentration from 1.2 mM to either 0.6 or 0.1 mM remarkably increased glucose-induced insulin secretion (GIIS) in primary islets isolated from C57BL/6 mice. Similarly, both the plasma insulin levels and GIIS rose in isolated islets of *Trpm6^Δ17 /fl^;Villin1-Cre* mice. We attribute these rises to augmented increases in intracellular Ca^2+^ oscillations in pancreatic β-cells. However, the glycemic metabolic profile was not impaired in *Trpm6^Δ17 /fl^;Villin1-Cre* mice, suggesting that chronic hypomagnesemia does not lead to insulin resistance. Collectively, the results of this study suggest that neither acute nor chronic Mg^2+^ deficiency suppresses glucose-induced rises in insulin secretion. Even though hypomagnesemia can be symptomatic of T2D, such deficiency may not account for declines in insulin release in this disease.

## 1. Introduction

Hypomagnesemia (serum Mg^2+^ levels < 0.7 mmol/l) has been reported in various cohorts of patients with type 2 diabetes (T2D) [1,2]. Notably, intracellular free Mg^2+^ levels are also significantly lower in these patients than in nondiabetic cohorts. However, the underlying relationship between Mg^2+^ deficiency and the pathophysiological processes of diabetes remains unclear. It is uncertain whether Mg^2+^ deficiency plays a role in triggering the onset of diabetes or if it instead occurs as a consequence of the development of the diabetic condition in patients.

There is substantive evidence suggesting that Mg^2+^ promotes β-cell function and insulin secretion [3,4]. Increases in blood glucose levels stimulate the influx of glucose into pancreatic β-cells via GLUT2, followed by a glucokinase-mediated conversion into glucose-6-phosphate (G6P). Mg^2+^ directly increases glucokinase activity by acting as a cofactor for adenine nucleotides [5,6]. G6P is further metabolized through glycolysis and the Krebs cycle, resulting in increased ATP levels. These rises inhibit K_ATP_ channel activity, which depolarizes the membrane potential difference and increases intracellular Ca^2+^ influx through stimulating voltage-dependent calcium channel (VDCC) activity. This final step induces rises in insulin release from β-cells [7]. The activity of K_ATP_ is tightly controlled by a delicate balance between the opposing effects of changes in intracellular ATP and MgADP levels, which inhibit and stimulate its activity, respectively [8,9,10]. Despite the established role of Mg^2+^ in controlling β-cell function, the impact of hypomagnesemia on glucose-induced insulin secretion (GIIS) is unclear.

A recent study demonstrated that short-term exposure to low Mg^2+^ concentrations stimulates GIIS in murine islets [11]. Although we confirmed this positive effect of acute hypomagnesemia on insulin secretion, it is notable that diabetic patients usually experience chronic hypomagnesemia [12]. To the best of our knowledge, the effect of chronic hypomagnesemia on insulin secretion was only studied in the rodent models fed a Mg^2+^-deficient diet rather than in any genetic model of this condition. In this current study, we used a mouse model of hypomagnesemia to investigate the effect of chronic Mg^2+^ deficiency on β-cell function. These studies were undertaken to assess if long-term exposure to hypomagnesemia induces decreases in insulin secretion in diabetic patients.

Over the last decade, different genetic forms of hypomagnesemia have been detected in humans. They include mutations in transient receptor potential melastatin type 6 (TRPM6), Paracellin-1, and cyclin M2 (CNNM2) [13,14,15]. Loss-of-function mutations in TRPM6 cause hypomagnesemia in human infants [14,16,17]. A recent study demonstrated that deletion of *Trpm6* in mice intestines results in hypomagnesemia, reduced bone Mg^2+^ content, and diminished urinary Mg^2+^ excretion [18]. The Mg^2+^ levels in the serum of intestine-specific *Trpm6* KO mice were only 50% of the control values. In this study, to delineate the effects of chronic hypomagnesemia, we used 24-week-old *Trpm6^Δ17 /fl^;Villin1-Cre* mice in which *Trpm6* is selectively deleted in the intestine [18]. Furthermore, TRPM6 is expressed in the distal convoluted tubule (DCT) of the kidney and facilitates luminal Mg^2+^ uptake from the pro-urine [17]. Such expression in the DCT is critical for maintaining whole-body Mg^2+^ balance [19]. Therefore, we additionally used the *Trpm6^Δ17 /fl^;Ksp-Cre* mouse model in which *Trpm6* is selectively deleted in the kidney. Our results indicate that both short-term and long-term Mg^2+^ deficiency have a positive effect on insulin secretion from murine pancreatic islets. We found that hypomagnesemia induced rises in insulin secretion through the enhancement of oscillatory Ca^2+^ responses in pancreatic β-cells.

## 2. Materials and Methods

### 2.1. Islet Isolation and Determination of Insulin Secretion

All experimental procedures were approved and performed in accordance with the EU Animal Welfare Act and were approved by the District Government of Upper Bavaria, Germany. C57BL/6 mice were obtained from The Jackson Laboratory. *Trpm6^Δ17 /fl^;Villin1-Cre KO* and *Trpm6^Δ17 /fl^*;*Ksp-Cre* mice, both with C57BL/6J background were reported previously [18]. Islets were isolated from 8- to 24-week-old male and female mice on a chow diet. Isolation of pancreatic islets was performed as previously described [20]. In brief, the pancreas was perfused by injection of 3 mM Collagenase-P (Roche, Mannheim, Germany) (0.3 mg/mL) in Hank’s buffered salt solution (HBSS) containing 25 mM HEPES and 0.5% (*w*/*v*) BSA into the common bile duct. Isolated islets were recovered for 48 h in RPMI 1640 (Thermo Fisher Scientific, Germany), supplemented with 1% penicillin/streptomycin (P/S) and 10% fetal bovine serum (FBS), in humidified 5% CO_2_, at 37 °C. After this period, islets were used for functional assessments. Before determination of insulin secretion, islets were equilibrated for 1 h in KRB-Buffer (115 mM NaCl; 4.5 mM KCl; 1.2 mM KH_2_PO_4_; 2.6 mM CaCl_2_; 1.2 mM MgCl_2_; 10 mM HEPES; 20 mM NaHCO_3_; 0.1% (*w*/*v*) BSA; pH 7.4) with 2.8 mM glucose. To assess the acute effect of Mg^2+^ deficiency on insulin secretion, islets were preincubated for 3 or 24 h in three different Mg^2+^ concentrations (1.2, 0.6, and 0.1 mM MgCl_2_) in KRB solution supplemented with 5 mM glucose and 0.1% (*w*/*v*) BSA. The Mg^2+^ concentrations of KRB solution during the GIIS were adjusted accordingly to match the Mg^2+^ concentrations used during the preincubation period. To assess the chronic effect of Mg^2+^ deficiency on insulin secretion, isolated islets from *Trpm6^Δ17 /fl^;Villin1-Cre* and *Trpm6^Δ17 /fl^*;*Ksp-Cre* mice were maintained in the 0.6 mM Mg^2+^ condition. Accordingly, GIIS was also performed in KRB buffer containing a 0.6 mM Mg^2+^ concentration, and the effects of 20 mM glucose on insulin secretion were monitored in the low-Mg^2+^ condition (0.6 mM). Determination of insulin secretion from the islets was performed in 12-well plates containing 600 μL KRB (8 islets/well, at least 5 independent experiments performed in triplicates). After the aforementioned preincubation time, islets were incubated for 1 h in either 2.8 or 20 mM glucose. Released insulin was measured in the supernatant using an insulin ELISA kit (ALPCO, Salem, MA, USA).

### 2.2. Calcium Imaging

Islets were loaded with 4 µM fluo-4 AM (Invitrogen, Waltham, MA, USA) for 2 h at room temperature in extracellular buffer containing 138 mM NaCl; 5.6 mM KCl; 2.6 mM CaCl_2_; 1 mM MgCl_2_; 5 mM HEPES; pH 7.4 [21]. Changes in [Ca^2+^]_i_ were recorded by laser scanning confocal microscopy using an LSM 510 Meta system (Zeiss, Jena, Germany) in conjunction with a water immersion objective (63X/NA1.2). Individual cells were selected as “regions of interest” with the LSM software (Zen2.3), and their calcium responses to the different stimuli were measured as alterations in fluo-4 emission intensity at 500–550 nm upon excitation with the 488-nanometer line of an argon laser, 8-bit 512 × 512 pixels images were acquired every 5 s.

### 2.3. Calculation of Calcium Oscillation Frequency and Amplitude

The calculation of calcium oscillation was previously described [21]. In brief, the algorithm is based on three stages that parse over an array of data (the “input data array” or IDA). First stage is marking all areas of the IDA in a second array (the “rising slope indicator array” or RSIA). For each value in the IDA, if the current value in the IDA is higher or equal to the previous value, then the input data is rising and will be marked in the RSIA with a value higher than zero (10 in this case to be more visible in chart). If the current value in the IDA is lower than the previous value, then the data is falling, and it will be marked with a 0 in the RSIA. Second stage involves interrogating the areas in the RSIA where a value greater than zero is found and writing the results in a third array (the “accumulator array” or AA). For each value in the RSIA, if the value is greater than zero, then the difference between the current value of the IDA and the previous value of the IDA, and the previous value of the AA are written in the current AA position. The third stage is used to indicate where the peaks are in the data in a fourth array (the “peak array” or PA). For each value in the RSIA, if the current value of the RSIA is smaller than the previous value of the RSIA then this was the peak, and the current value of the RSIA is written in the PA. If the current value of the RSIA is larger than the previous value of the RSIA, then the peak has not yet been reached, and the value zero will be written in the PA.

### 2.4. Characterization of Glucose Homeostasis

For investigation of blood parameters, samples were collected after euthanasia using EDTA-coated microvette tubes (Sarstedt, Nümbrecht, Germany), immediately cooled on ice, centrifuged at 2000× *g* and 4 °C for 10 min, and plasma stored at −80 °C. Plasma insulin was quantified using an Insulin ELISA assay (ALPCO, Salem, MA, USA). Blood glucose levels were measured by a glucometer (TheraSense FreeStyle).

### 2.5. Statistics

Data are expressed as mean ± S.E.M. A *p* value less than 0.05 was considered significant. Graph presentations, curve fittings, statistics, and *p* values were obtained using Prism software (version 9.0.1; GraphPad, San Diego, CA, USA). For comparison of two groups, *p* values were calculated by the unpaired two-tailed Student’s *t*-test for parametric or Mann–Whitney test for non-parametric distribution. For 3 groups, one-way ANOVA with Bonferroni’s multiple comparison was used for parametrically distributed data.

## 3. Results

### 3.1. Lowering Extracellular Mg^2+^ Concentrations Enhances GIIS in Murine Islets

**Figure 1:**
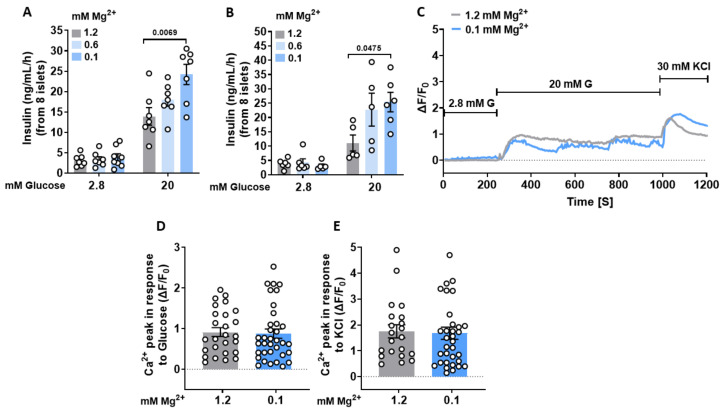
Lowering extracellular Mg^2+^ concentrations increases GIIS in murine islets. Islets were isolated from 8-week-old mice. (**A**,**B**) Insulin secretion was measured in intact wild-type (*n* = 8, from 5 mice) after 3 h (**A**), and (*n* ≥ 5, from 6 mice) after 24 h (**B**) of incubation with three different Mg^2+^ concentrations (1.2, 0.6, and 0.1 mM) in the presence of 2.8 or 20 mM glucose. (**C**) Intact wild-type islets (*n* ≥ 20, from 5 mice) were loaded with 4 µM fluo-4 AM, and alterations in [Ca^2+^]_i_ of individual cells were monitored using confocal microscopy after increasing the extracellular glucose concentration from 2.8 to 20 mM in the presence of 1.2 mM or 0.1 mM Mg^2+^. KCl (30 mM) was used as a positive control. Mean traces for 1.2 mM and 0.1 mM Mg^2+^ are shown in gray and blue, respectively. (**D**,**E**) Average Ca^2+^ influx peaks assessed from baseline after glucose (**D**) and KCl (**E**) stimulation in wild-type β-cells in the presence of 1.2 or 0.1 mM Mg^2+^ (*n* ≥ 20, from 5 mice). The cells that displayed no increase in [Ca^2+^]_i_ in response to high glucose concentration are excluded from the results. Data are shown as means ± S.E.M. (circles in bar graphs represent single values) and statistical differences were assessed by one-way ANOVA (**A**,**B**) or unpaired two-tailed Student’s *t*-test (**D**,**E**). *p* value is shown above the bar.

We assessed the short-term effect of low extracellular Mg^2+^ concentration on insulin secretion, after 3 h and 24 h preincubation with either 1.2, 0.6, or 0.1 mM MgCl_2_. Each of these declines in the Mg^2+^ concentration dose-dependently increased GIIS in pancreatic islets isolated from C57BL/6 mice. Increases in the glucose concentration from 2.8 to 20 mM enhanced insulin exocytosis about 4.5-fold in islets in the presence of a physiological concentration of Mg^2+^ (1.2 mM), whereas 3 h preincubation with 0.6 and 0.1 mM Mg^2+^ concentrations increased insulin exocytosis 5.3-fold and 6.2-fold, respectively (Figure 1A). Moreover, twenty-four-hour preincubation with either 0.6 or 0.1 mM MgCl_2_ increased insulin exocytosis nine-fold and eight-fold, respectively (Figure 1B).

### 3.2. Lowering Extracellular Mg^2+^ Concentrations Augments Ca^2+^ Oscillatory Amplitudes in Murine Islets

**Figure 2:**
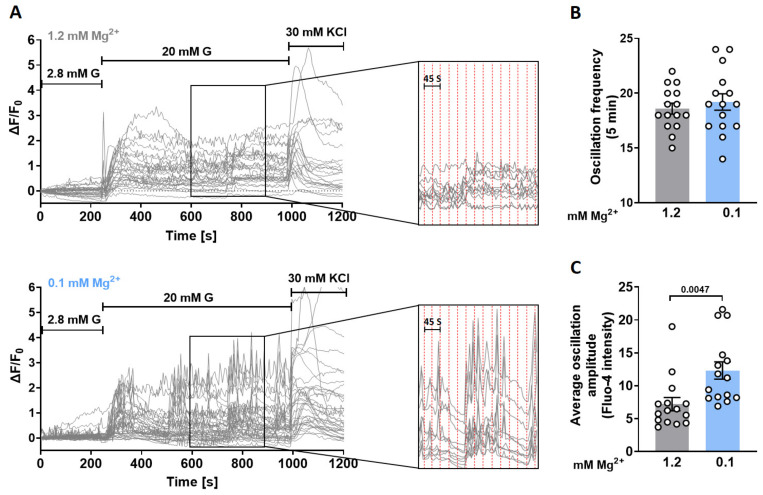
Lowering extracellular Mg^2+^ concentrations increases Ca^2+^ oscillatory amplitudes in murine islets. Islets were isolated from 8-week-old mice. (**A**) Intact wild-type islets (*n* ≥ 15, from 5 mice) were loaded with 4 µM fluo-4 AM, and alterations in [Ca^2+^]_i_ of individual cells were monitored using confocal microscopy after increasing the extracellular glucose concentration from 2.8 to 20 mM in the presence of 1.2 mM or 0.1 mM Mg^2+^. KCl (30 mM) was used as a positive control. Inserts show amplified sections indicating the regions from which data in the right panels were taken. (**B**,**C**) Comparison of oscillation frequency and average oscillation amplitudes in the presence of 1.2 or 0.1 mM Mg^2+^. Data are shown as means ± S.E.M. (circles in bar graphs represent single values) and statistical differences were assessed by unpaired two-tailed Student’s *t*-test (**B**,**C**). *p* value is shown above the bar.

To determine if exposure to a low Mg^2+^ concentration enhanced GIIS through increases in Ca^2+^ signaling, laser scanning confocal microscopy was used to monitor these Ca^2+^ responses in individual cells in intact islets. Twenty mM glucose rapidly induced [Ca^2+^]_i_ transients that were similar to one another in the presence of either 1.2 mM or 0.1 mM Mg^2+^ (Figure 1C,D). The Ca^2+^ transient patterns induced by 30 mM KCl under identical conditions were also remarkably similar, suggesting that canonical K_ATP_ signaling is not affected by different Mg^2+^ concentrations (Figure 1E). Nevertheless, despite the comparable oscillation frequency (Figure 2A,B), the average oscillatory amplitudes were significantly larger in the presence of 0.1 mM than those in the presence of 1.2 mM Mg^2+^ (Figure 2A,C). This difference prompted us to hypothesize that a low Mg^2+^ concentration underlies the enhanced oscillatory responses and increased GIIS.

### 3.3. Plasma Levels of Insulin and GIIS Rise in Intestine-Specific Trpm6 KO Mice

**Figure 3:**
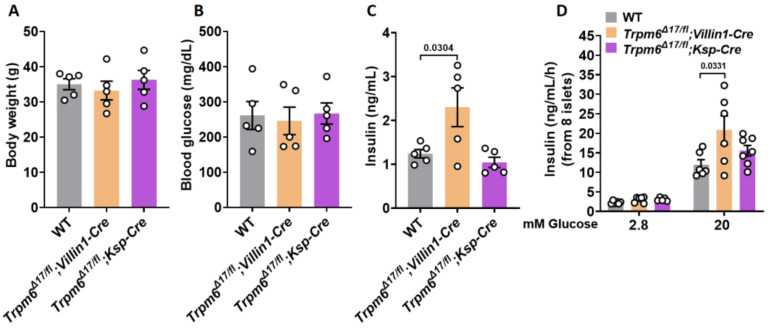
Tissue-specific *Trpm6* deletion in intestine increases plasma levels of insulin and GIIS. (**A**) Body weight, (**B**) blood glucose levels, and (**C**) plasma insulin levels (*n* = 5) in 24-week-old male and female *Trpm6^Δ17 /fl^;Villin1-Cre*, *Trpm6^Δ17 /fl^;Ksp-Cre*, and wild-type mice on standard chow diet. (**D**) Insulin secretion (ng/mL/h/8 islets) in isolated islets of 24-week-old male and female *Trpm6^Δ17 /fl^;Villin1-Cre*, *Trpm6^Δ17 /fl^;Ksp-Cre*, and wild-type mice. Islets were incubated for 1 h in the presence of low glucose (2.8 mM) or high glucose (20 mM) (*n* = 5 mice per genotype). Data are shown as means ± S.E.M., and statistical differences were assessed by one-way ANOVA. Circles in bar graphs represent single values. *p* values are shown above the bars.

To assess the effects of long-term hypomagnesemia on insulin secretion and glucose homeostasis, we determined if the loss of *Trpm6* function in the intestine (*Trpm6^Δ17 /fl^;Villin1-Cre*) and kidney (*Trpm6^Δ17 /fl^;Ksp-Cre*) of mice alters glucose metabolism. The body weights of these two different genotypes that were fed a standard chow diet were similar to their wild-type counterpart (Figure 3A). Although the blood glucose concentrations remained unaffected in all three genotypes (Figure 3B), the plasma insulin levels were significantly higher in the *Trpm6^Δ17 /fl^*;*Villin1-Cre* mice (Figure 3C). Furthermore, a marked increase in GIIS was detected in isolated islets from *Trpm6^Δ17 /fl^;Villin1-Cre* mice. An increase in the glucose concentration from 2.8 to 20 mM enhanced insulin release about 5-fold in wild-type islets, whereas basal insulin exocytosis rose 6.8-fold in *Trpm6^Δ17 /fl^;Villin1-Cre* islets and 5.4-fold in *Trpm6^Δ17 /fl^;Ksp-Cre* islets (Figure 3D). Collectively, these data show that chronic hypomagnesemia in *Trpm6^Δ17 /fl^;Villin1-Cre* mice induces increases in insulin secretion, which we hypothesize are attributable to increases in insulin exocytosis. Importantly, the glycemic metabolic profile was not impaired in *Trpm6^Δ17 /fl^;Villin1-Cre* mice, indicating that chronic hypomagnesemia in this mouse model does not result in severe insulin resistance. Nevertheless, if we had access to a larger sample size, it would strengthen the robustness and reliability of these findings.

### 3.4. Intestine-Specific Trpm6 KO Islets Exhibit an Increased Ca^2+^ Oscillation Amplitude

**Figure 4:**
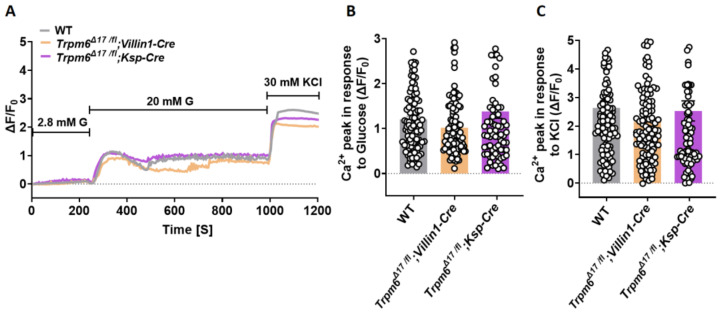
Tissue-specific *Trpm6* deletion in intestine and kidney has no effect on glucose- and KCl-induced Ca^2+^ transients in murine islets. Islets were isolated from 24-week-old mice. (**A**) Intact WT (*n* ≥ 100, from 5 mice), *Trpm6^Δ17 /fl^;Villin1-Cre* (*n* ≥ 100, from 5 mice), and *Trpm6^Δ17 /fl^;Ksp-Cre* (*n* ≥ 100, from 5 mice) islets were loaded with 4 µM fluo-4 AM, and alterations in [Ca^2+^]_i_ of individual cells were monitored using confocal microscopy after increasing the extracellular glucose concentration from 2.8 to 20 mM. KCl (30 mM) was used as a positive control. Mean traces are shown for every mouse model. (**B**,**C**) Average Ca^2+^ influx peaks assessed from baseline after glucose (**B**) and KCl (**C**) stimulation in WT, *Trpm6^Δ17 /fl^;Villin1-Cre*, and *Trpm6^Δ17 /fl^;Ksp-Cre* β-cells. The cells that displayed no increase in [Ca^2+^]_i_ in response to a high glucose concentration are excluded from the results. Data are shown as means ± S.E.M. (circles in bar graphs represent single values). Statistical differences were assessed by one-way ANOVA.

Twenty mM glucose induced similar Ca^2+^ transients in islets isolated from *Trpm6^Δ17 /fl^;Villin1-Cre*, *Trpm6^Δ17 /fl^;Ksp-Cre*, and control littermates (Figure 4A,B). Furthermore, KCl (30 mM) induced similar responses under identical conditions in all three genotypes (Figure 4C).

**Figure 5:**
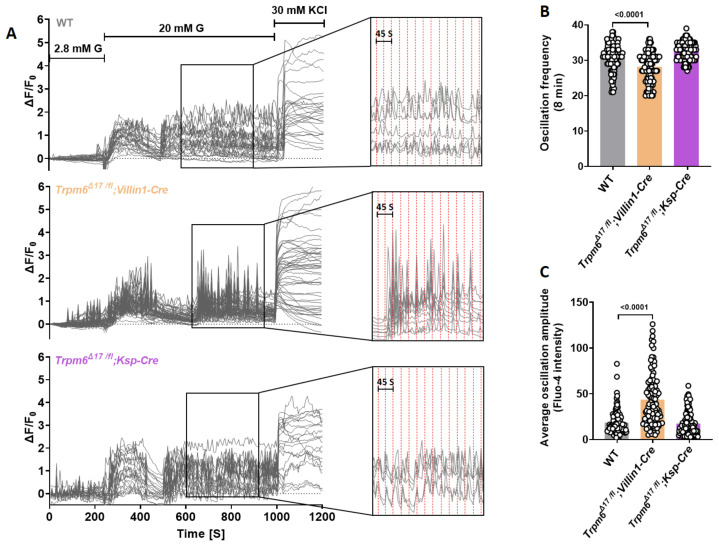
Tissue-specific *Trpm6* deletion in intestine increases Ca^2+^ oscillation amplitude. Islets were isolated from 24-week-old mice. (**A**) Intact wild-type (*n* ≥ 100, from 5 mice), *Trpm6^Δ17 /fl^;Villin1-Cre* (*n* ≥ 100, from 5 mice), and *Trpm6^Δ17 /fl^;Ksp-Cre* (*n* ≥ 100, from 5 mice) islets were loaded with 4 µM fluo-4 AM, and alterations in [Ca^2+^]_i_ of individual cells were monitored using confocal microscopy after increasing the extracellular glucose concentration from 2.8 to 20 mM in the presence of 1.2 mM or 0.1 mM Mg^2+^. KCl (30 mM) was used as a positive control. Inserts show amplified sections indicating the regions from which data in the right panels were taken. (**B**,**C**) Comparison of oscillation frequency and average oscillation amplitudes between intact wild-type, *Trpm6^Δ17 /fl^;Villin1-Cre*, and *Trpm6^Δ17 /fl^;Ksp-Cre* islets. Data are shown as means ± S.E.M. (circles in bar graphs represent single values) and statistical differences were assessed by one-way ANOVA. *p* values are shown above the bar.

Nevertheless, *Trpm6^Δ17 /fl^;Villin1-Cre* islets showed a remarkable alteration in oscillatory responses relative to control islets. They displayed a diminished oscillation frequency (Figure 5A,B) and an elevated average oscillation amplitude during the plateau phase (Figure 5C). This association between the loss of *Trpm6* function and augmentation of the oscillatory response suggests that Mg^2+^ concentration affects mechanisms underlying extracellular insulin exocytosis.

## 4. Discussion

Overt clinical hypomagnesemia is particularly common in T2D patients afflicted with poorly controlled glycemic profiles [22]. Dietary Mg^2+^ supplementation is reported to be beneficial in various clinical studies in diabetic patients [23,24]. However, the cause-and-effect relationship is unclear between hypomagnesemia and this disease. Although clinical studies suggested that there is a positive correlation between Mg^2+^ concentration and insulin secretion [25], in vivo and in vitro experimental data disagree with this notion.

A recent study demonstrated that exposure of wild-type islets to a low extracellular Mg^2+^ concentration increased insulin exocytosis when the islets were cultured for 24 h under a high glucose condition that mimicked the T2D hyperglycemic condition [11]; furthermore, knockdown of transient receptor potential melastatin 7 (*Trpm7*) channels, the most abundant Mg^2+^ channel in β-cells, increased GIIS in rat insulinoma cell line (INS-1) [11]. A similar inverse relationship exists in another rat insulinoma cell line (RIN m5F), in which the depletion of extracellular Mg^2+^ stimulates insulin secretion [26,27]. Our results confirmed this inverse relationship between declines in intracellular Mg^2+^ concentration and increases in insulin secretion and linked them to increases in peak amplitudes of Ca^2+^ oscillation in pancreatic β-cells.

Even though we found that short-term Mg^2+^ deficiency does not impair insulin secretion, this finding might not contravene the relationship between hypomagnesemia and T2D since diabetic patients usually experience chronic hypomagnesemia. Thus, we hypothesize that impaired insulin secretion in diabetic patients may be attributed to long-term Mg^2+^ deficiency in these individuals. An earlier study demonstrated that rats fed a Mg^2+^-deficient diet for 6 weeks did not show any overt phenotype; however, after a period longer than 6 weeks on this diet, glucose tolerance and GIIS both improved in isolated islets relative to the control [28]. On the contrary, another study indicated an impaired glucose tolerance in rats fed a Mg^2+^-deficient diet for 6 weeks [29]. Consistent with this finding, glucose tolerance was improved in Zucker Diabetic Fatty (ZDF) rats after 6 weeks of Mg^2+^ supplementation [30].

In this current study, we used genetically modified animal models to assess the effects of long-term hypomagnesemia on β-cell function in mice. Recently, we generated tamoxifen-inducible β-cell-specific *Trpm7* KO mice (β*Trpm7* KO) and monitored the metabolic phenotype of this mouse model within 28 weeks. Notably, GIIS was severely diminished in isolated islets from β*Trpm7* KO mice after 28 weeks of tamoxifen-induced recombination [21]. Here, we hypothesized that the progressive impairment in insulin secretion might be attributable to the chronic Mg^2+^ deficiency in β-cell-specific *Trpm7* KO mice; however, this difference may instead stem from the fact that TRPM7 is a bifunctional protein consisting of a serine/threonine protein kinase linked to the channel moiety [31,32]. Importantly, we recently demonstrated the indispensable role of the kinase moiety of TRPM7 in mediating the expression of key β-cell markers and the major transcription factors involved in insulin biosynthesis and maintaining β-cell identity [21]; furthermore, TRPM7 can also function as a Ca^2+^ channel [33]. Hence, β*Trpm7* KO mice might not be a suitable mouse model for delineating the role of Mg^2+^ in insulin secretion and β-cell function. We took another approach to evaluate if there is an association between hypomagnesemia and β-cell function. Here, we used an established mouse model of hypomagnesemia with organismal Mg^2+^ deficiency known as *Trpm6^Δ17 /fl^;Villin1-Cre* [18]. Our results indicate that conditional *Trpm6* inactivation in the intestine increased the plasma levels of insulin. Nevertheless, the body weight and blood glucose levels remain similar among the *Trpm6^Δ17 /fl^;Villin1-Cre* and the control littermates. Furthermore, the high glucose condition in isolated *Trpm6^Δ17 /fl^;Villin1-Cre* pancreatic islets increased insulin secretion to levels that were higher than in the wild-type. This result is in agreement with an observation in a Canadian T2D cohort in which HOMA-β, an index of functional pancreatic β-cell mass, was inversely correlated with serum Mg^2+^ levels [34].

Mg^2+^ homeostasis is regulated by Mg^2+^ reabsorption from pro-urine in DCT [19]. Given the critical role of TRPM6 in controlling Mg^2+^ reabsorption [17], we characterized the metabolic phenotype of a conditional *Trpm6* KO in the kidney (*Trpm6^Δ17 /fl^;Ksp-Cre*). Our result demonstrated that *Trpm6* inactivation in the kidney had no impact on body weight, blood glucose, and plasma levels of insulin.

An increase in intracellular Ca^2+^ influx is the final trigger of insulin exocytosis. We found that chronic hypomagnesemia increased the average amplitudes of the Ca^2+^ oscillatory responses in *Trpm6^Δ17 /fl^;Villin1-Cre* islets. Here, we suggest that Mg^2+^ deficiency attenuates intracellular levels of MgADP in β-cells. Since the K_ATP_ channel activity is dependent on a balance between ATP-mediated suppression (at Kir6.2) and MgADP activation (at SUR1), a decline in MgADP content enhances ATP-induced K^+^ channel inhibition. Furthermore, Mg-nucleotides increase the number of functional K_ATP_ channels in the plasma membrane [35]. Consequently, a Mg^2+^ deficiency prolongs the depolarization phase in β-cells, resulting in larger increases in the amplitudes of Ca^2+^ oscillations and ultimately enhancing insulin release. Importantly, rises in MgADP content have been shown to reduce the interburst intervals [10]. Thus, we conclude that chronic hypomagnesemia reduced the MgADP content and, in turn, reduced the Ca^2+^ transient oscillation frequency in *Trpm6^Δ17 /fl^;Villin1-Cre* islets.

Various studies have demonstrated that declines in both intracellular and extracellular Mg^2+^ inhibit L-type Ca^2+^ channel activity [36,37]; therefore, it can be speculated that reduced intracellular Mg^2+^ concentrations due to hypomagnesemia increase L-type channel activity that, in turn, increases Ca^2+^ oscillatory amplitudes. However, it has also been reported that long-term hypomagnesemia instead reduces the expression of L-type Ca^2+^ channels and reduces Ca^2+^ responses [38]. Nevertheless, these findings were solely obtained in cardiomyocytes rather than in pancreatic β-cells.

Continuous exposure to increased insulin levels downregulates plasma membrane insulin receptor expression, which in turn augments insulin resistance [39]; however, we did not obtain any evidence suggesting that prolonged exposure to elevated insulin levels downregulates insulin receptor expression. This is supported by the absence of any detectable impairment in the metabolic phenotype of *Trpm6^Δ17 /fl^;Villin1-Cre* mice. This negative effect rules out a major impediment in insulin sensitivity in this mouse model of chronic hypomagnesemia.

## 5. Conclusions

The role of Mg^2+^ in controlling insulin secretion has only been investigated in a limited number of clinical studies [25,34]; furthermore, the beneficial effect of Mg^2+^ supplementation on β-cell function in individuals without diabetes was only observed in a small randomized clinical trial [40]. Hence, in contrast to current thinking, our findings strongly suggest that long-term hypomagnesemia does not negatively impact the metabolic phenotype, insulin secretion, and β-cell function. Accordingly, it is more likely that hypomagnesemia is the consequence of diabetes rather than a causative factor. One possible explanation for the association between hypomagnesemia and T2D is that insulin plays a vital role in controlling the reabsorption of Mg^2+^ in the kidney through modulating TRPM6 channel activity. The increased renal excretion of Mg^2+^ induced by a decrease in insulin may contribute to the development of hypomagnesemia in individuals with T2D [41].

## Data Availability

The data that support the findings of this study are available on request from the corresponding author.
